# Visual simulators replicate vision with multifocal lenses

**DOI:** 10.1038/s41598-019-38673-w

**Published:** 2019-02-07

**Authors:** Maria Vinas, Clara Benedi-Garcia, Sara Aissati, Daniel Pascual, Vyas Akondi, Carlos Dorronsoro, Susana Marcos

**Affiliations:** 1 Institute of Optics, Spanish National Research Council, IO-CSIC, Serrano, 121, Madrid, 28006 Spain; 2 Present Address: Department of Ophthalmology, Stanford University, Palo Alto, California USA

## Abstract

Adaptive optics (AO) visual simulators based on deformable mirrors, spatial light modulators or optotunable lenses are increasingly used to simulate vision through different multifocal lens designs. However, the correspondence of this simulation with that obtained through real intraocular lenses (IOLs) tested on the same eyes has not been, to our knowledge, demonstrated. We compare through-focus (TF) optical and visual quality produced by real multifocal IOLs (M-IOLs) -bifocal refractive and trifocal diffractive- projected on the subiect’s eye with those same designs simulated with a spatial light modulator (SLM) or an optotunable lens working in temporal multiplexing mode (SimVis technology). Measurements were performed on 7 cyclopleged subjects using a custom-made multichannel 3-active-optical-elements polychromatic AO Visual Simulator in monochromatic light. The same system was used to demonstrate performance of the real IOLs, SLM and SimVis technology simulations on bench using double-pass imaging on an artificial eye. Results show a general good correspondence between the TF performance with the real and simulated M-IOLs, both optically (on bench) and visually (measured visual acuity in patients). We demonstrate that visual simulations in an AO environment capture to a large extent the individual optical and visual performance obtained with real M-IOLs, both in absolute values and in the shape of through-focus curves.

## Introduction

Adaptive Optics (AO), a technology originally developed to image stellar objects with ground-based telescopes eliminating the degrading effects of the atmospheric turbulence^1^, has more recently expanded applications into microscopy^2^ and ophthalmology^3,4^. Fundus cameras and scanning laser ophthalmoscopes provided with AO have allowed imaging of individual photoreceptor cells and microscopic structures in the retina. Conversely, AO has allowed probing the visual system under manipulated optics^5–8^, either with fully corrected optical aberrations^9^, through the optical aberrations of another subject or scaled versions of their own^10,11^, or a phase pattern simulating a given correction (i.e. an intraocular lens, contact lens or a corneal treatment)^5,12^.

AO visual simulators are particularly attractive to test vision in patients with new optical designs^6,13^ prior to delivering surgical corrections to the patient or even manufacturing the lenses. Simulations of new corrections with adaptive optics primarily serve to investigate interactions between the patient’s optics and a given correction, to investigate differences across corrections, and eventually to select the correction that optimizes perceived visual quality and performance in patients^5,12,14^.

Providing patients the visual experience before implanting an intraocular lens or fitting a contact lens is particularly relevant for multifocal corrections for presbyopia (the age-related loss of the ability to dynamically focus near and far objects)^15^. Multifocal corrections work under the principle of simultaneous vision, projecting simultaneously focused and defocused images on the retina. These corrections generally provide multifocality at the expense of reducing optical quality at all distances. There are several multifocal designs, working on refractive or diffractive principles, including refractive bifocal concentric or angular designs, diffractive bifocal and trifocal designs, and extended depth of focus designs with smooth refractive profiles or hybrid refractive-diffractive designs^16^. Visual simulators allow undertaking systematic studies of visual performance testing multiple lens designs (programmable in the adaptive optics active element), which can be directly compared by the patient. As clinical instruments, AO visual simulators can help demonstrating the patient the experience of multifocality and can guide the patient and eye care practitioner in the selection of the most suitable correction.

In AO-based visual simulators, an active optical element (deformable mirror, spatial light modulator, or optotunable lens) reproduces the equivalent phase map of a certain optical design in a plane conjugate to the subject’s pupil plane, while the observer is looking at a visual stimulus. Deformable Mirrors (DM) allow simulating smooth optical designs, or to induce certain amounts of aberrations, while controlling the aberrations of the subject. DMs have been used, for example, to evaluate the effects of inducing spherical aberration^17,18^, or combinations of astigmatism and coma on through-focus (TF) visual performance^19^. In contrast, spatial light modulators (SLMs)^5,20–22^, generally liquid crystal-based on silicon (LCoS)-SLMs devices, are capable of reproducing abrupt phase maps due to their high spatial resolution, and to increase the effective phase range through the use of wrapped phase representations^23,24^. In prior work, we have studied perceived visual quality at far, intermediate and near distances with SLMs simulating bifocal, trifocal and tetrafocal, angular and radially segmented corrections^5,12,25^. Other studies have also used SLMs to simulate the effect of corneal inlays^26^ and to map diffractive optics^9,27^. Reflective-DM or SLM-based visual simulators are mostly limited to experimental environments, given their relatively high complexity and dimensions, although some have made their way into commercial products^28,29^. In these devices the visual experience is limited to stimuli projected in a display, subtending a relatively small (typically <2 degree) visual field, in many cases monocularly^3^.

Ideal visual simulators in a clinical environment should be see-through, allowing a direct view of the real world and should display a larger visual field. Visual simulators of bifocal corrections, with two optical channels superimposing two images using a transmission SLM to simulate different pupillary masks, have been used on clinical subjects^13^, but still remain in a laboratory setting. Deformable multi-actuator lenses have been recently released, which may be suitable to reproduce smooth surface-varying multifocal optics, although, to our knowledge, they have not been yet used in visual simulators^30^, and won’t be capable of mapping diffractive or segmented optics. An interesting novel approach to simultaneous vision simulation is the use of optotunable lenses working in temporal multiplexing mode, a technology developed by our group (SimVis technology), described in detail in previous publications^14,31^. The tunable lens scans multiple foci to provide superimposed images on the retina, all of them with the same position and magnification, but corresponding to different planes in focus. These custom electronically driven lenses can produce fast periodic foci variations at speeds greater than the flicker fusion threshold of the human visual system, delivering seemingly static images on the subject’s retina that emulate the effect of the multifocal correction. The simulation of multifocal corrections relies on evaluating the TF energy distribution of the correction, from the knowledge of the spatially varying pupillary power distribution, and programming in the optotunable lens the corresponding time-varying focus changes. The simulated multifocal correction is tuned to match the TF optical quality (in terms of Visual Strehl)^32^ of real existing multifocal lenses. It is an optimization of the electrical input signal driving the tunable lens and, consequently, of the SimVis technology TF optical quality^31^. Real multifocal intraocular lens (M-IOL) designs are therefore temporally mapped by evaluating the corresponding temporal profile of the optical power of a tunable lens that results in a TF optical quality matching the TF optical quality of the M-IOL. While symmetric MIOL designs can be fully captured using a temporal pattern, some limitations are expected for asymmetric complex designs.

The goal of this study is to compare on real subjects, for the first time to our knowledge, TF optical and visual quality produced by real M-IOLs and visual simulations of those multifocal designs using two different active optical elements, a spatial light modulator (SLM) and temporal multiplexing with optotunable lenses (SimVis technology), all of them incorporated in a polychromatic AO Visual Simulator.

## Results

TF optical quality (double-pass aerial retinal point images and E-letter stimulus images, on-bench) and visual acuity (VA), in 7 patients, were measured with two M-IOLs: a bifocal refractive segmented IOL, Bi-R, and a trifocal diffractive IOL, Tri-D. Those corrections, of complex design, were tested in a polychromatic AO visual simulator for 3 different conditions: the real lens, simulations in a SLM, and simulations using SimVis technology. All measurements were performed monocularly, in green light (555 nm) and for 5-mm pupils.

### On-bench tests

**Figure 1:**
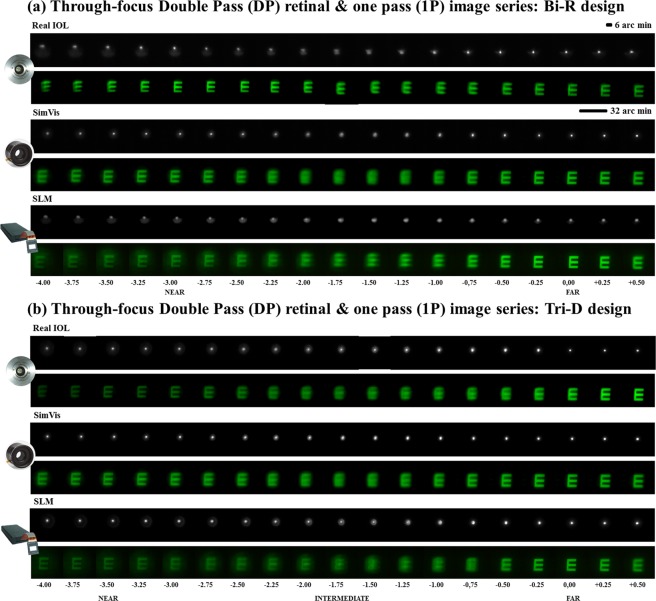
TF Optical quality on-bench testing. On-bench TF Double-pass (DP) aerial retinal point images and TF retinal images of an E-optotype (1P) through the bifocal refractive (**a**) and the trifocal diffractive (**b**) designs for all simulated conditions. Scale bars account for the angular extent of the images (6′ for the DP and 32′ for the 1P images).

Figure 1(a,b) shows TF double-pass (DP) aerial images and E-letter images (1P) obtained on-bench with the three simulating conditions, real IOL, SimVis technology and SLM, for (a) bifocal refractive segmented lens, Bi-R, and (b) the trifocal diffractive lens, Tri-D. Qualitatively, the replication of the images with the simulators is highest around the foci for both simulators. Also, the asymmetric bifocal design (Bi-R) produces an asymmetry in the PSF (reminiscent of vertical coma^33^), which is apparent with real IOL and SLM, but cannot be reproduced with SimVis.

**Figure 2:**
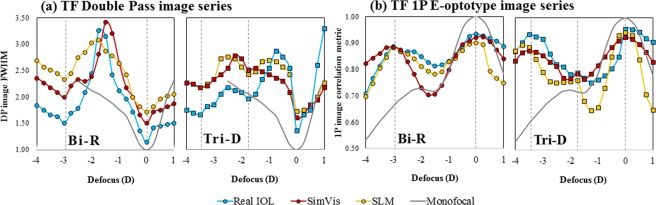
TF optical quality metrics. (**a**) TF double-pass optical quality (FWHM) for the bifocal refractive IOL (Bi-R) and the Trifocal diffractive IOL (Tri-D), gray line, for a monofocal lens (no IOL) as a reference; (**b**) TF image correlation metric, gray line, for a monofocal lens (no IOL) as a reference. Blue symbols represent the real IOL; Red symbols represent the SimVis technology simulation and yellow symbols represent the SLM Simulations.

Figure 2 shows TF optical quality metrics obtained from the on-bench images series: (a) full width at half-maximum (FWHM) for the double-pass images and (b) image correlation metric for the TF E-letter images, with the different multifocal designs (Blue lines: real IOL; red lines: SimVis; yellow lines: SLM)). In addition, the TF curve obtained from on-bench image series for a monofocal condition (no multifocal design) are shown (grey lines) for the TF DP and the 1P image series. TF DP images were normalized to the 0 D monofocal image series, so that FWHM = 1 at 0.0 D for the monofocal curve, while 1P image series were correlated to the image of 0.0 D of the monofocal TF range, where image correlation was 1 for the monofocal image at 0.0 D. There is a good correspondence between both the double-pass images and 1P image series across all simulating conditions in the position of best near and far focus (Bifocal: 0.0 D for far vision, +3.00 D addition for near vision; Trifocal: 0.0 D for far vision, +1.75 D addition for intermediate vision and +3.50 D addition for near vision).

**Figure 3:**
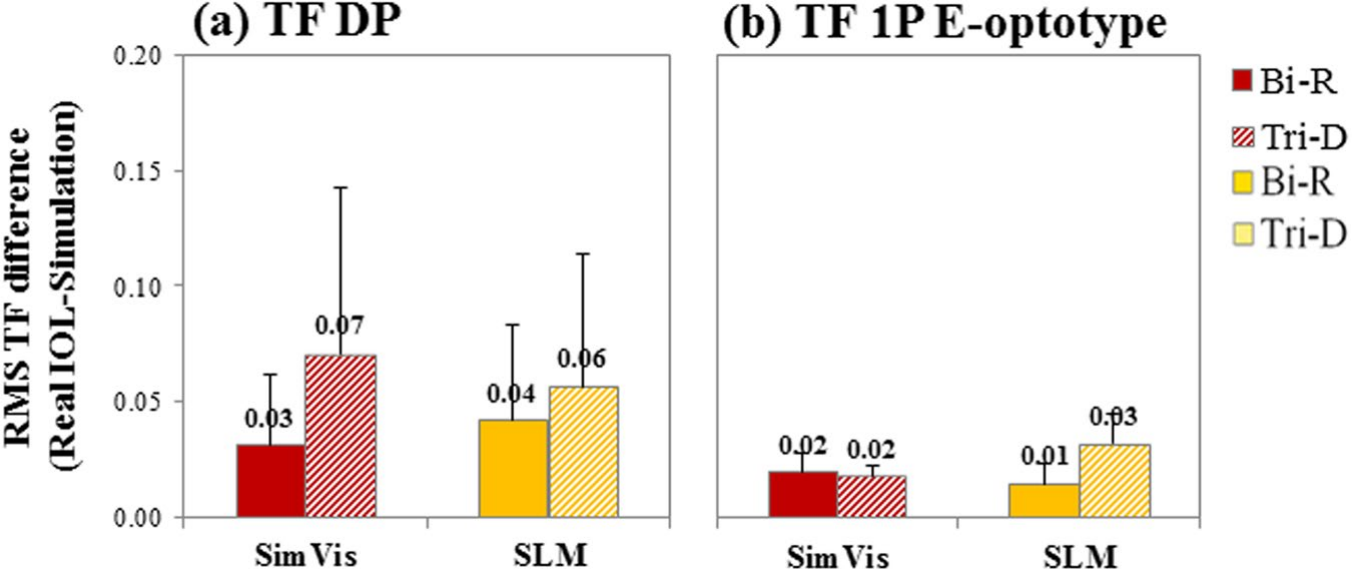
Comparison of TF optical quality metrics. RMS TF difference of the TF curves (5.0-D range) with respect to the real multifocal IOL, for SimVis technology (red bars) and SLM (yellow bars) (**a**) for TF Double-Pass curves, and (**b**) for TF 1P E-optotype image correlation curves. Solid bars are for the bifocal refractive IOL (Bi-R); Shaded bars are for the Trifocal diffractive IOL (Tri-D). Data are for 5-mm pupils.

RMS TF difference in the TF curves (5-D range) between the real multifocal IOL and the simulation was taken as a metric for the quality of the simulation. Figure 3 compares the RMS TF difference for SimVis technology (red bars) and SLM (yellow bars) for the two analyzed optical quality metrics: TF DP aerial retinal image curves (a) –data from Fig. 2a, and for TF 1P E-optotype image (b) correlation curves –data from Fig. 2b. In both cases, the RMS TF difference is below 0.07, and as low as 0.01-0.02 in some conditions. When comparing both simulating techniques, the RMS TF difference between SLM and SimVis technology TF curves is statistically significant only for Tri-D design with both TF optical quality metrics (paired-samples t-test: TF 1P, t = 2.70, p = 0.014; TF DP, t = −2.90, p = 0.008). When comparing both designs, the RMS TF difference between Bi-R and Tri-D TF curves is significantly different for SLM (TF 1P; paired-samples t-test: t = −4.10, p = 0.01) and for SimVis technology (TF DP; paired-samples t-test: t = −2.40, p = 0.025), while there is no significant differences between them for SimVis technology (TF 1P) and SLM (TF DP).

### Through focus visual acuity

**Figure 4:**
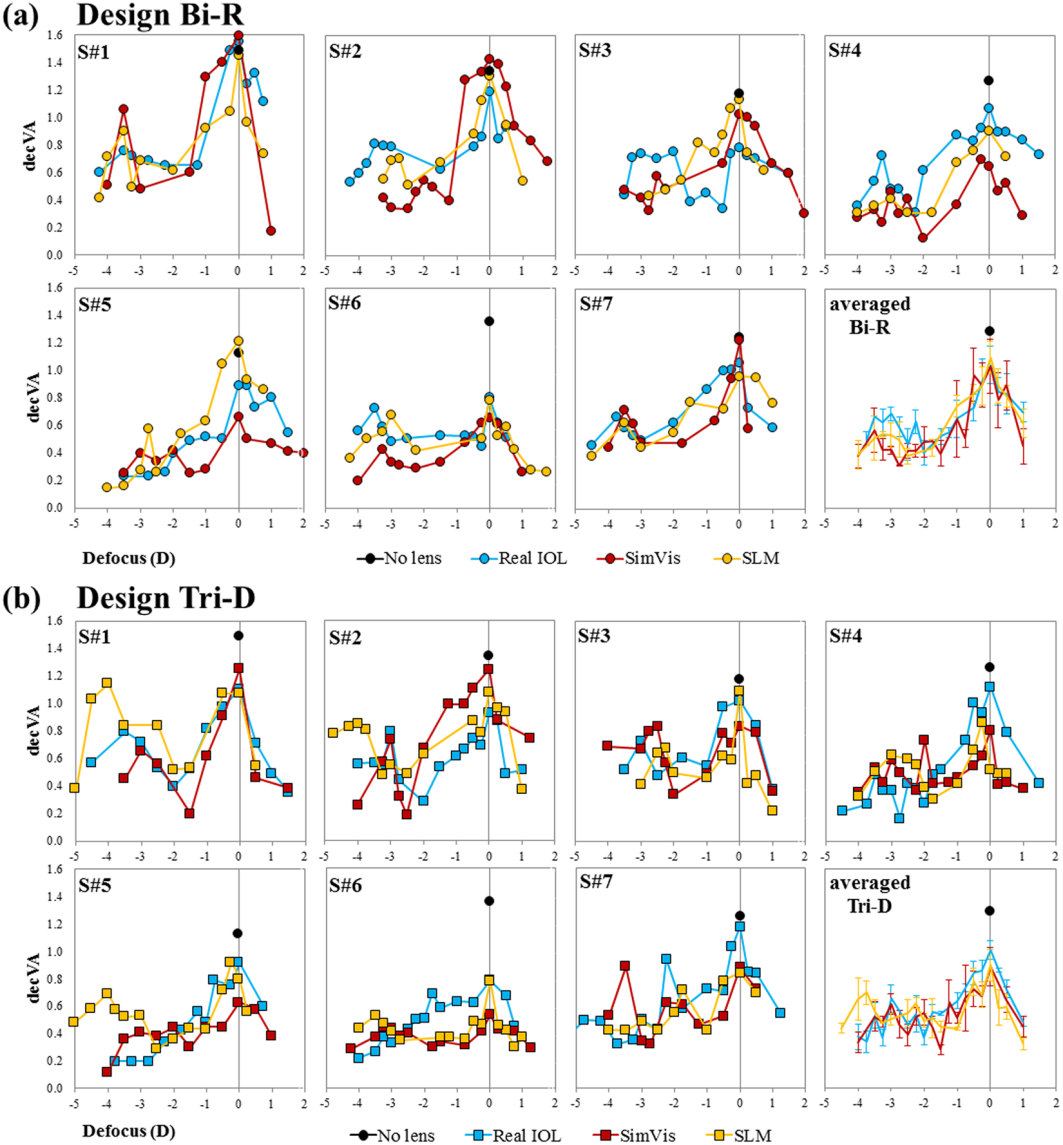
TF decimal VA on patients. TF decimal VA for all 7 subjects and all conditions (no lens, black dot; real IOL, blue line; SimVis technology, red line; SLM, yellow line) for the two simulated designs: (**a**) Bi-R (circles) and (**b**) Tri-D (squares). Averaged data across subjects is shown for both designs. Error bars stands for inter-subject deviation.

Figure 4 shows the TF decimal VA for the 7 subjects participating in the study and the 4 conditions measured (no lens, real IOL, SimVis technology, and SLM) for the two simulated designs: (a) Bi-R and (b) Tri-D in a 5.00 D range. The last plot in each panel represents the averaged data across subjects (bottom, right. VA measurements are highly repeatable (averaged standard deviation: 0.03 ± 0.005). TF VA curves showed individual similarity across simulations (either using SimVis technology or SLM) and real IOLs in all 7 subjects. VA obtained through the real IOLs correlated statistically with those obtained through SimVis technology (r = 0.71 and r = 0.46; p < 0.05 across subjects, for Bi-R and Tri-D, respectively) and SLM (r = 0.46 and r = 0.56; p < 0.05 across subjects for Bi-R and Tri-D, respectively). Averaged data showed a good agreement between the TF curves. A mixed model analysis for repeated measurements was performed to investigate differences in outcomes for the two simulation techniques in comparison with Real IOLs TF performance, for both designs (Bi-R & Tri-D). The analysis showed no significant differences for any of the simulators when using as factors the TF performance and the simulator for both designs (Bi-R p = 0.911 & Tri-D p = 0.504), indicating that while there may be differences between the curves point by point, the general shape of the TF curves is preserved.

**Figure 5:**
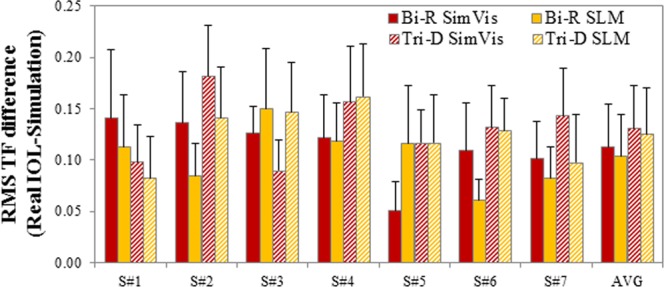
Comparison of TF VA across designs. RMS difference of TF VA curves (5.0-D range) for all subjects with respect to the real multifocal IOL, for SimVis technology (red bars) and SLM (yellow bars). Solid bars are for the bifocal refractive IOL (Bi-R); Shaded bars are for the Trifocal diffractive IOL (Tri-D). Error bars stand for inter-subject variability. Data are for 5-mm pupils.

Figure 5 shows the RMS TF difference between the TF curves for the real IOL and both SimVis technology and SLM. The average RMS TF difference of the simulated Bi-R design with respect to the real Bi-R IOL was 0.11 ± 0.02 for the SimVis technology & 0.11 ± 0.02 for the SLM. The average RMS TF difference for Tri-D was 0.13 ± 0.016 for the SimVis technology & 0.13 ± 0.02 for the SLM, respectively. The differences across simulators are not statistically different (paired-samples t-test: Bi-R, t = −0.81, p = 0.46; Tri-D, t = −0.45, p = 0.67). The differences between both designs (Bi-R and Tri-D) for real IOLs and the different simulations are statistically different for the SimVis technology (paired-samples t-test: t = −2.29, p = 0.04), but not for the SLM.

On average, RMS TF difference in subjects was higher for Tri-D than for Bi-R, in line with results from the on-bench measurements previously described. On the other hand, different multifocal designs produce different TF performances on the same subject (average RMS TF difference of 0.11 ± 0.01, 0.14 ± 0.04, and 0.12 ± 0.02 for real IOLs, SimVis technology and SLM, respectively between the two IOL designs).

## Discussion

AO visual simulators based on different active optical elements are increasingly used to simulate vision through different multifocal lens designs. However, the correspondence of this simulation with the vision obtained through the physically manufactured real IOLs tested on the same eyes had not been, to our knowledge, demonstrated. In this study, we compared for the first time TF optical and visual quality produced by real M-IOLs with visual simulations using a spatial light modulator (SLM) or an optotunable lens working in temporal multiplexing mode (SimVis technology), in monochromatic light. We found a general good correspondence between the through-focus performance with the real and simulated M-IOLs, both optically (on bench) and visually (measured VA in patients).

We did not find a bias for higher reproducibility of the TF performance towards a particular type of simulator, although the very different principles of operation may favor one or the other depending on the design of the lens or the stimuli. For example, the temporal patterns for SimVis technology are programmed using the TF Visual Strehl performance of the theoretical lens as a target, and the results on patients demonstrate that it captures adequately the TF visual performance (and the optical image quality using a FWHM metric). However, as SimVis technology is limited to represent symmetric patterns, the blur produced on the images is invariably symmetric, which may be the reason for the discrepancies in the appearance of the E-optotype images (particularly those between foci) in the SimVis technology simulation (showing symmetric, more degrading, blur) compared to the real IOL or the SLM (where the asymmetric blur appears to be less noticeable) Fig. 1(a)^31^. Also, measurements were performed monochromatically. While SimVis technology is not affected by chromatic aberration and the temporal patterns could be programmed to modify the effects of chromatic aberration on the TF visual Strehl curve that serves as a template for SimVis technology, SLMs are largely affected by chromatic artifacts^34^, as the phase map is in fact only representative of one single wavelength. This is of great importance in novel diffractive M-IOL designs, where chromatic aberration is used to generate the multifocal component of the lens^35^, thus the SLM pattern and SimVis technology signal would need to be modified accordingly. Since capturing the specific chromatic effects of the diffractive lenses pose a challenge in SLM-based simulators, it is likely that measurements with polychromatic stimuli (instead of the green stimuli as used in this study) would result in lower performance for the SLM.

Our results also support the use of visual simulators in the clinic. TF performance with the same IOL largely varies across individuals, indicating that the visual experience of a multifocal correction is rather unique to the patient and therefore valuable to be demonstrated to a patient prior to implantation. We found lower differences in TF performance across patients with the same IOL (0.10 ± 0.02 RMS TF difference) than in different IOLs on the same patient (0.13 ± 0.02 RMS TF difference). Visual simulators can help identifying those patients whose visual quality will be largely affected by a multifocal correction. For example, patient S#6 experiences a large drop in VA with both multifocal corrections (44.6% for the Bi-R and 48.5% for the Tri-F compared to the monofocal performance at far), and while depth-of-focus is enlarged, VA remains low for a large range. On the other hand, most patients experience minimal changes in VA for far (average multifocal VA/monofocal VA at far, 1.05 for the Bi-R and 0.95 for the Tri-F) and exhibit functional VA at a near (average VA for near, 0.60 for the Bi-R and 0.53 for the Tri-F), and even an intermediate peak/range for the Tri-F.

The significant differences in TF performance of the same IOL across subjects are likely associated to the different interactions between the subjects native aberrations and the IOL optics^5,6^, and to a lesser extent, to neural factors and adaptation of the subject to native aberrations. While in the current study, measurements were done under natural aberrations (and these were not included as a variable in the study) it is interesting to note that the AO instrument in this study allows measurement and correction of these aberrations. An interesting open question is whether the TF performance would have been more similar across subjects had the native aberrations of the eye been corrected. On the other hand, despite the contribution of the subject’s aberrations to the effective TF, there are clear observable features in the TF curves attributable to the lens design. For example, TF VA with the trifocal IOL in patient S#7 reveals clearly three best foci (both with the real and simulated IOL). The ability of visual simulators to capture the performance of the specific IOL designs supports their clinical utility not only to demonstrate multifocality to a prospective patient, but also to demonstrate differences across different commercial lenses.

The ultimate utility of the visual simulators relies on their application on patients prior to intraocular lens implantation. The tested intraocular lenses are designed to replace the natural crystalline lens. Visual simulators are designed generally to be used on phakic eyes, while a post-operative validation of the real IOL will not include the contribution of the crystalline lens (except for phakic IOLs). As the cornea is the major contributor to the ocular aberrations we expect the crystalline lens contribution to pre-operative measurements to be secondary, particularly in the presence of a multifocal correction. Furthermore, a cataractous crystalline lens will produce an overall decrease of visual performance. While a direct pre- and post-operative comparison of TF visual quality with simulators and real IOL is only possible for clear crystalline lens, we expect (particularly with zonal segmented corrections) SimVis technology to be generally less affected by opacities, as due to the temporal multiplexing the simultaneous image will be projected on the retina bypassing opacities^31^.

The current study demonstrates that visual simulations in an AO system capture to a large extent the optical and visual performance obtained with real IOLs, both in absolute values and the shape of TF curves when compared, for the first time, on the same individual patients. Visual simulators based on different technologies (real IOLs, SLMs, SimVis technology) are useful programmable tools to predict visual performance with M-IOLs.

## Methods

TF optical and visual quality, produced by real M-IOLs, was compared with visual simulations using two different active optical elements, a phase-only reflective LCoS-SLM and SimVis technology (temporal multiplexing of an optotunable lens), in a custom-made polychromatic Adaptive Optics (AO) Visual Simulator. Vision was tested on seven cyclopleged subjects, through two real multifocal IOLs (bifocal refractive and trifocal diffractive) projected on the subject’s pupil plane and their corresponding simulations with SLM and SimVis technology. The experiments were performed in a multi-channel polychromatic AO visual simulator, with the DM correcting the optical aberrations of the system. All channels shared a similar display where the visual stimuli were shown. The same system was used to demonstrate performance of the real IOLs, SLM and SimVis technology simulations on bench, using double-pass retinal imaging and an artificial eye.

### AO-based visual simulator

On-bench measurements and visual tests were performed in a custom-developed polychromatic AO system at the Visual Optics and Biophotonics Lab (Institute of Optics, Spanish National Research Council, IO-CSIC, Madrid, Spain), described partially in previous publications^5,8,12^. In this AO system, the visual stimulus is seen through 3 different active optical elements: (1) a reflective deformable mirror (DM), used in this study to correct the subject and the system aberrations; (2) a reflective phase-only spatial light modulator (LCoS-SLM), and (3) a simultaneous vision simulator (SimVis technology) based on temporal multiplexing of a opto-tunable lens, both used to simulate the multifocal designs; and for the purposes of the current study, real multifocal IOLs placed in a cuvette. The DM, SLM, SimVis technology and real IOLs were placed in conjugate pupil planes of the system.

**Figure 6:**
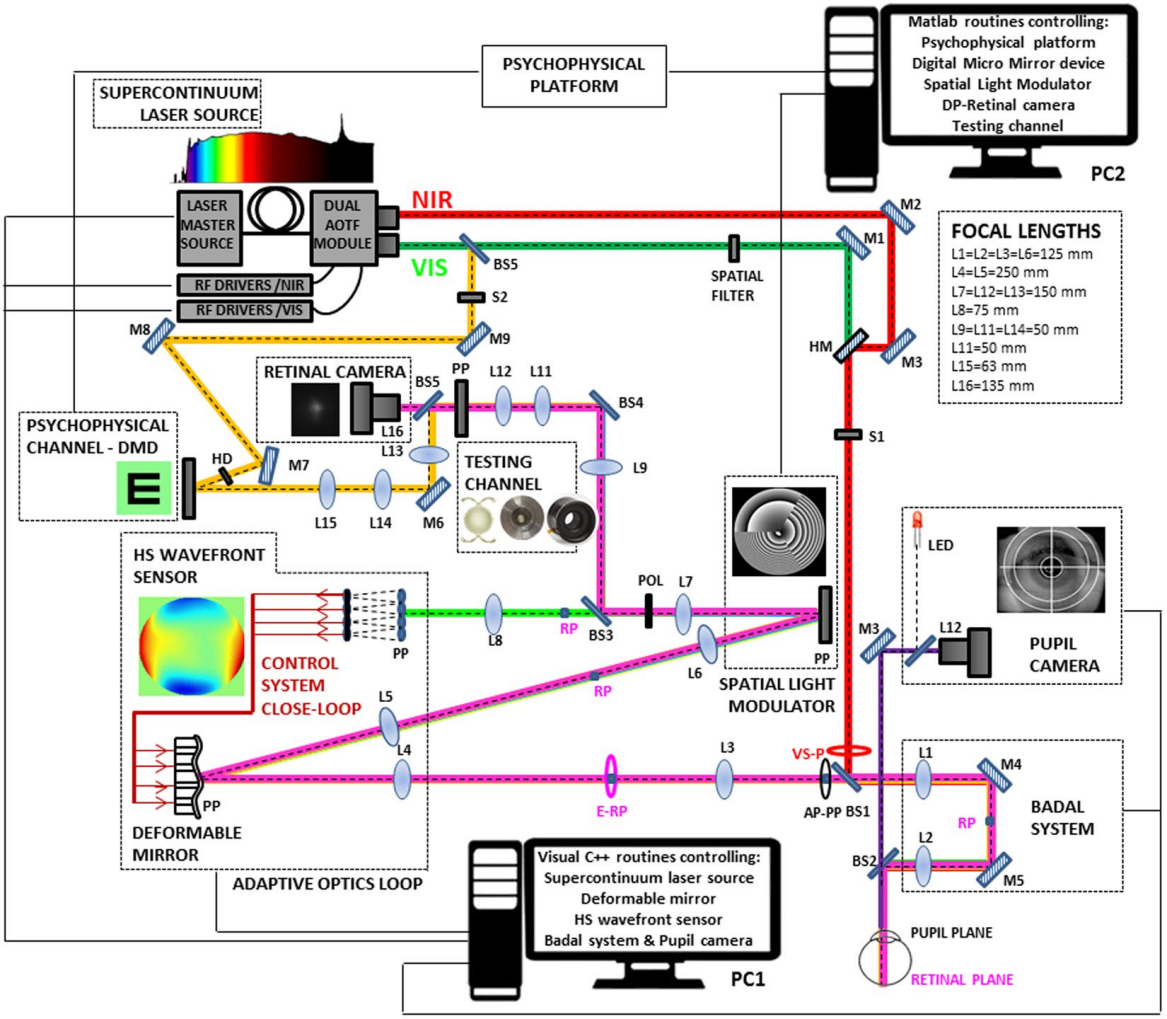
Polychromatic AO visual simulator. Schematic diagram of the custom-made polychromatic adaptive-optics system (VioBio Lab AO II system) with the 8 different channels in its current configuration (October, 2017): (1) the Illumination-Channel (red line); (2) the AO-Channel (green line); (3) the SLM-Channel (yellow line); (4) the Testing-Channel (blue line); (5) the retinal imaging-Channel (pink line); (6) the Psychophysical-Channel (orange line), (7) the Pupil Monitoring-Channel (purple line), and (8) the Badal optometer-Channel. NIR: near infrared light; VIS: visible light; RP: retinal plane; PP: pupil plane; BS: beam splitter; S: shutter; L: lens; M: mirror; HM: hot mirror; POL: polarizer; E-RP: retinal pinhole; AP-PP: artificial pupil; VS-P: variable size pupil.

The current configuration of the system, shown in a schematic diagram in Fig. 6, is formed by 8 different channels:

1. The *Illumination-Channel*, contains a supercontinuum laser source (SCLS, SC400 femtopower 1060 supercontinuum laser, Fianium Ltd, United Kingdom), in combination with a dual acousto‐optic tunable filter (AOTF) module (Gooch & Housego, United Kingdom), operated by RF drivers, to automatically select the wavelengths in the different channels (visible 450–700 nm or near infrared light 700–1100 nm, in our system configuration). The output is a collimated beam coupled to two independent multimode fibers. Illumination coming from the two independent fiber-channels of the SCLS enters the system collinearly by means of a hot mirror (HM), allowing wavefront sensing and retinal aerial imaging with visible (VIS) and near infrared (NIR) light. The 2-mm diameter beam entering the eye is slightly (1 mm) decentered with respect to the pupil center to avoid corneal reflections in the Hartmann-Shack images. Illumination coming from the VIS multimode fiber (BS5) is also used to monochromatically illuminate the visual stimuli. In this study, a visible wavelength (555 nm) was used to illuminate the visual display. Aberration and double-pass retinal images were collected in NIR (827 nm). The laser power measured at the corneal plane ranged between 0.5 and 50 μW, which was one order of magnitude below the ANSI standards safety limits at all tested wavelengths^36^.
2. The *AO-Channel* consists of a Hartmann-Shack wavefront sensor (microlens array 40 × 32, 3.6 mm effective diameter, centered at 1062 nm; HASO 32 OEM, Imagine Eyes, France) and an electromagnetic deformable mirror (DM) (52 actuators, 15-mm effective diameter, 50-µm stroke; MIRAO, Imagine Eyes, France), to measure and correct subjects and system aberrations, respectively.
3. The *SLM-Channel* that consists of a reflective phase-only LCoS-SLM (SLM; VIS; Resolution: 1920 × 1080; Pixel pitch: 8.0 µm; Holoeye Photonics AG, Germany) is used to generate the multifocal designs.
4. The *Testing-Channel*, placed in a conjugate pupil plane of the system, allows evaluating the simultaneous vision simulator (SimVis technology), as well as, the real IOLs. A custom-developed cuvette was used to place the real IOLs, which was composed by two assembled metal pieces with transparent windows and an internal support to set the real IOL in the proper plane and orientation. Expansion joints provided watertight seal so that the cuvette can be filled with distilled water. The DM, the wavefront sensor, the SLM and the testing plane (real IOLs and SimVis technology) are conjugate to the pupil by different relays of lenses. Magnification from the pupil is 2x to the DM, 1x to the SLM and the testing plane, and 0.5x to the wavefront sensor.
5. The *Retinal imaging-Channel* allows capturing retinal images of a 250 µm point source, and consists of a CCD camera (Retiga 1300, CCD Digital Camera, 12-bit, Monochrome, 6.7 × 6.7 µm pixel size, 1024 × 1280 pixels; QImaging, Canada), a collimating lens (L9, 50-mm focal length) and a camera lens (L16, 135-mm focal length) in the double-pass retinal imaging channel. The laser beam is filtered before entering the eye by means of a spatial filter (Fig. 6) composed of a microscope objective (20x), a 25 μm-pinhole and a 50 mm lens. This channel acts in fact as a “one-and-a half pass”, with the aerial image being the autocorrelation^37^ of the image of the laser spot with a 2-mm entry beam and that with a 1-mm exit beam.
6. The *Psychophysical-Channel*, placed in a conjugate retinal plane, consists of a Digital Micro-Mirror Device (DMD) (DLP® Discovery™ 4100 0.7 XGA, Texas Instruments, USA), and allows displaying visual stimuli with a 1.62 deg angular subtend. The DMD is monochromatically illuminated with light coming from the SCLS, 555 nm in this experiment. A holographic diffuser (HD) placed in the beam path breaks the coherence of the laser beam providing a uniform illumination of the stimulus.
7. The *Pupil Monitoring-Channel*, which allows monitoring of pupil size and subject position during measurements, consists of a camera (DCC1545M, High Resolution USB2.0 CMOS Camera, Thorlabs GmbH, Germany) conjugated to the eye’s pupil (by means of a 105-mm focal length objective lens (L12)).
8. The *Badal optometer-Channel* corrects for defocus in AO-, SLM-, Testing- and Psychophysical-Channels and allows TF psychophysical testing. Two automatized shutters allow simultaneous illumination of the eye (S1) and the stimulus (S2).

All optoelectronic and mechanical elements of the AO set-up were automatically controlled and synchronized using custom-built software (Visual C++ and C# (Microsoft). The custom-developed routines make use of the manufacturer’s Software Development Kit for Hartmann-Shack centroiding detection and wave aberration polynomial fitting.

### Multifocal designs: Bifocal refractive and Trifocal diffractive

**Figure 7:**
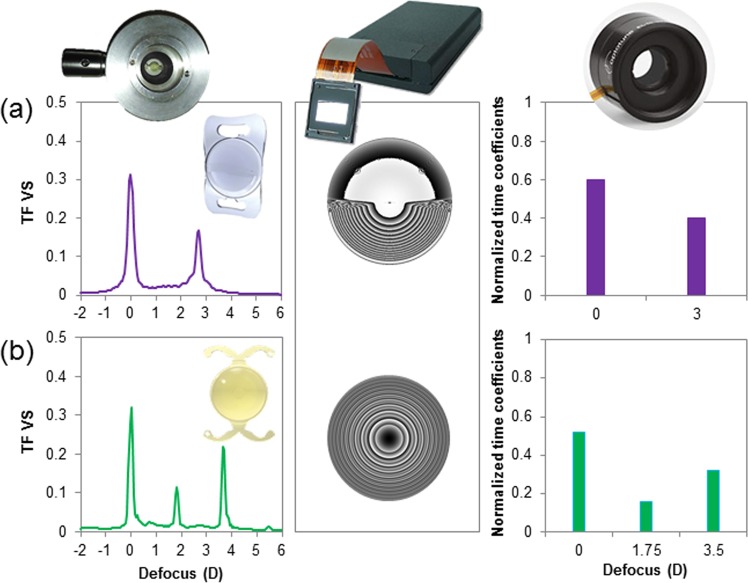
Multifocal designs evaluated in the study in terms of Visual Strehl (VS): (**a**) Bifocal non-rotationally symmetric refractive, Bi-R (MPlus, Oculentis); and (**b**) Trifocal diffractive, Tri-D (POD F FINeVision, PhysIOL), in the form of real IOLs (left), phase maps on an SLM (center) and temporal profile on an optotunable lens working on temporal multiplexing mode (right).

Two M-IOLs (bifocal non-rotationally symmetric refractive, Bi-R, and trifocal diffractive, Tri-D) were projected on the eye’s pupil, and also mapped in the SLM (as a spatial phase map) and on SimVis technology (as a temporal profile), shown in Fig. 7.

The bifocal non-rotationally symmetric refractive (Bi-R) IOL design mimics the Lentis MPlus LS-313 MF30 (Oculentis, Berlin, Germany), a multifocal acrylic refractive IOL, made out of hydrosmart, a copolymer consisting of acrylates with hydrophobic surface, UV absorbing (n = 1.46). The optical design consists of an aspherical surface with a posterior sector shaped near vision segment, which provides 2 useful focal distances: 0.0 D for far-vision, and +3.00 D addition for near-vision.

The trifocal diffractive (Tri-D) design corresponds to the POD F (FINeVision, PhysIOL, Liege, Belgium), a hydrophilic (26% hydrophilic acrylic) aspheric multifocal diffractive IOL built with a combination of two bifocal diffractive patterns, of which one is for far and near-vision and the other for far and intermediate-vision^38,39^. The combination of the two diffractive structures provides 3 useful focal distances: 0.0 D for far-vision, +1.75 D addition for intermediate-vision and +3.50 D addition for near-vision^38^.

### Real IOLs

Two 0-D M-IOLs with the Bi-R and Tri-D designs (provided by the manufacturers) were inserted in a cuvette filled with distilled water, placed in a conjugate pupil plane, and projected on the eye’s pupil of the subjects. Calculated TF curves (Fig. 7, right plots) show distinct bifocal and trifocal performance.

### Spatial light modulator (SLM)

The multifocal phase maps (Bi-R and Tri-D) were extracted in pseudophakic computer eye models from the knowledge of the surface height profiles of the lenses, provided by the respective manufacturers, as described in a previous publication^33^. Matlab routines were used to numerically simulate the multifocal phase designs, the Bi-R and Tri-D, which were later programmed in a reflective phase-only LCoS-SLM. The SLM addressable phase maps (Φ) were evaluated from the multifocal phase maps by performing 2π-wrapping such that, Φ = X [mod 2π], where X represents the unwrapped phase map. and Φ is the wrapped phase map. The generated phase pattern is a grey-scale image, where each level of grey corresponds to a certain phase difference in the interval [0 2π] (Fig. 7, central plots). The images were generated for a 5-mm pupil.

### SimVis technology & Temporal multiplexing

Both multifocal designs were mapped using SimVis technology with a temporal profile, as shown in Fig. 7. The TF optical quality of the two multifocal designs in terms of Visual Strehl (VS)^32^ was estimated for a 5 mm pupil diameter at 555 nm. The corresponding SimVis temporal profile that provides an equivalent TF VS was determined^31^. The temporal profiles were addressed with SimVis technology as shown in Fig. 7, right plots.

### On-bench testing

TF optical quality for the three conditions, real IOLs, SLM and SimVis technology, was evaluated on bench in the same AO system using TF double-pass retinal images and TF retinal images of an E-optotype from an artificial eye. Focus shifts were achieved by moving a Badal optometer from +1.00 D to −4.50 D in 0.25 D-steps, around the best foci for far.

*TF DP aerial retinal* images (DP) were obtained for an artificial eye in place of the human eye (consisting of a 50.8-mm focal length achromatic doublet lens and a rotating diffuser as an artificial retina) for the real IOL and the two simulations with the SLM and SimVis technology. Images of a “point-source” were obtained at 555 nm for a 5-mm pupil exit pupil diameter.

*TF retinal images of an E-optotype* (1.62-deg subtend) (1P) were collected on an artificial eye provided with an objective lens (50.8 mm) and a CCD camera (DCC1545M, High Resolution USB2.0 CMOS Camera, Thorlabs GmbH, Germany) acting as a “retina”, in place of the subject’s eye. The stimuli were displayed in the Digital Micro-Mirror Device (DMD), illuminated with 555 nm light from the SCLS, for 5-mm pupil diameter.

### Patients

Seven subjects were monocularly tested in the system under cycloplegia. Subjects were non-presbyopic (35 ± 3 years old) and nearly emmetropic (spherical error: −0.85 ± 0.90 D, astigmatism <0.50 D in all cases). The RMS for 3rd and higher order aberrations (5-mm pupil diameter) in the subjects ranged from 0.19 to 0.59 um.

All protocols met the tenets of the Declaration of Helsinki and had been previously approved by the Spanish National Research Council (CSIC) Bioethical Committee. All participants were acquainted with the nature and possible consequences of the study and provided written informed consent.

### Through-focus visual acuity

VA was measured using an 8-Alternative Forced Choice (8AFC)^40^ procedure with Tumbling E letters and a QUEST (Quick Estimation by Sequential Testing) algorithm programmed with the Psychtoolbox package^41^ to calculate the sequence of the presented stimulus (letter size and orientation) in the test following the subject’s response. Measurements were performed at different positions of the Badal Optometer ranging from −1.00 to +4.00 D, for the two lenses and the SLM and SimVis technology simulations. After looking for their best subjective focus without a multifocal correction and prior to measurements, subjects were shown the whole TF range with the corresponding design, so that they could identify the approximate position of their best focus for the different visual distances (far, intermediate and near). After that, measurements were performed at different positions of the TF range with higher sampling around the identified foci, which varied for each subject. The QUEST routine for each VA measurement consisted of 40 trials, each one presented for 0.5 seconds, where the threshold criterion was set to 75%. The threshold, VA measurement, was estimated as the average of the 10 last stimulus values. Visual acuity was expressed in terms of decimal acuity (logMAR = −log_10_[decimal acuity])^42^.

Variability of each VA measurement was obtained from the standard deviation of the 10 last stimulus values used to estimate the threshold in each measurement.

### Experimental protocol on patients

Subjects were stabilized using a dental impression and the eye’s pupil was aligned to the optical axis of the instrument (using an x-y-z stage moving a bite bar) using the line of sight as a reference, while the natural pupil is viewed on the monitor using a pupil camera. To ensure constant pupil diameter during the measurements, a 5-mm artificial pupil was placed in a conjugate pupil plane.

Measurements were performed monocularly, in a darkened room, under cycloplegia (by instillation of Tropicamide 1%, 2 drops 30 minutes prior to the beginning of the study, and 1 drop every 1 hour). The subject was asked to adjust the Badal system position to achieve best subjective focus. Subjects viewed the psychophysical stimulus generated by the Digital Micro-Mirror Device (DMD), illuminated monochromatically at 555 nm, through the real IOL, the SLM- or SimVis technology- simulated M-IOLs or the real IOLs and performed the corresponding psychophysical test (TF VA). The order of the simulation conditions was randomized. Subjects were instructed on the nature of the experiment and performed some trial runs prior to the test.

### Data analysis

TF optical quality was obtained from DP retinal images and images of the E-stimulus on bench. The double-pass image quality metric as defined as the full width at half maximum (FWHM) of the images^43^, so the width will be smaller for more focused images. Image series were obtained for each simulator under the same experimental conditions: laser power, pupil diameter, exposure time. However, due to the different baseline of the different technologies, exposure times were different across simulators and subsequently images were normalized to calculate the image quality metric, which was normalized to the 0 D image series within each TF image series.

The image quality metric for the E-stimulus was obtained from the correlation coefficient (correlation of the E-letter with a monofocal correction and each collected image in the same conditions: laser power, pupil diameter, exposure time) of the image series, after each image was centered. A sub-image of the best focused image from the monofocal image series was used in the correlation calculations

TF optical quality (on bench) and TF visual quality (in patients) curves were obtained for the real IOL and SLM and SimVis technology simulations for both the bifocal and trifocal lens designs. The comparison between the real and simulated performances were expressed in terms of Root-Mean-Square (RMS) difference of the linearly interpolated TF curves (in a 5.00 D range), taking the real IOL as the reference, as metric for the quality of the replication of the lens design by the simulators.

Statistical analysis was performed with SPSS software (IBM) to test differences across results with the different simulators, and optical designs (paired-samples t-test) in both cases: on-bench and on patients’ results (n = 7). A mixed model analysis for repeated measurements was performed to investigate differences in outcomes for the two simulation techniques, as a function of the two multifocal designs and the interaction with through focus level. In the model, fixed-effect factors were the TF steps of the TF VA curve, and the type of simulator, whereas the repeated effect was the combination of both. TF VA curve was the random effect factor in the model in all cases.

## Data Availability

All data generated or analyzed during this study are included in this published article. The datasets generated during and/or analyzed during the current study are available from the corresponding author on reasonable request.
